# Global Outbreak of Human Monkeypox in 2022: Update of Epidemiology

**DOI:** 10.3390/tropicalmed7100264

**Published:** 2022-09-25

**Authors:** Irena Ilic, Ivana Zivanovic Macuzic, Milena Ilic

**Affiliations:** 1 Faculty of Medicine, University of Belgrade, 11000 Belgrade, Serbia; 2 Department of Anatomy, Faculty of Medical Sciences, University of Kragujevac, 34000 Kragujevac, Serbia; 3 Department of Epidemiology, Faculty of Medical Sciences, University of Kragujevac, 34000 Kragujevac, Serbia

**Keywords:** human monkeypox, epidemiology, outbreak, risk factors

## Abstract

Background: Human monkeypox was a neglected zoonotic disease considered endemic to rainforests of rural parts of Central and Western Africa, until a global outbreak in May 2022. Methods: This review describes the epidemiological characteristics of human monkeypox. Results: Since the first confirmed case in the United Kingdom on 13 May 2022, and up until 19 September, more than 62,000 cases of human monkeypox were reported in 104 countries in the world (among them 97 countries where the monkeypox virus was not endemic). Up to today, 20 persons have died in this global outbreak. This outbreak predominantly affects men self-identifying as gay or bisexual or other men who have sex with men, and for now, there is no sign of continuous transmission of the disease in other populations. Today, the monkeypox outbreak is increasing alarmingly in many countries and presents a new challenge and a large issue for public health worldwide. The World Health Organization declared the global monkeypox outbreak a public health emergency of international concern on 24 July 2022. Before this outbreak, health professionals in many countries had a knowledge gap and a lack of experience in the management of monkeypox. Conclusions: Advances in the comprehension of the epidemiology of human monkeypox are necessary for effective prevention and outbreak response.

## 1. Introduction

While the COVID-19 pandemic is ongoing and the world experiences the consequences of the coronavirus 2019 disease, the globe has been jolted by the novel multi-country outbreak of human monkeypox [1,2,3,4,5]. In the global human monkeypox outbreak in 2022, the transmission of monkeypox seems to be highly unusual because for the first time the disease has been reported in countries that have not been affected before and have no epidemiological connection with countries that previously reported human monkeypox (such as the endemic countries in West and Central Africa) [5,6]. In this outbreak, no cases reported travel to Africa [6]. The World Health Organization (WHO) declared the global monkeypox outbreak a public health emergency of international concern on 24 July 2022 [7].

In recent weeks, a steep and rapid rise in the number of human monkeypox cases across the world has raised concerns [2,4]. About 100 countries have reported cases of monkeypox since May 2022, with over 62,000 confirmed cases [1]. Confirmed cases were mostly male (>99%), with a median age of 35–38 years (range: 18–67) [8,9].

In the ongoing large outbreak in non-endemic countries, the occurrence of almost all cases (>90%) is still identified primarily among men describing themselves as men who have sex with men [1,4,10,11]. In this outbreak, most of the human monkeypox cases have presented with mild to moderate symptoms [1,12,13,14,15]. The viruses were isolated from semen and vaginal fluids to confirm the possibility of their sexual transmission [16]. So far during this outbreak, there have been several cases of transmission to healthcare professionals via occupational exposure [1,17].

Previously, cases outside Africa were linked to international travel to endemic countries or were associated with imported animals [18,19]. Even though the natural monkeypox virus animal reservoir is still unidentified, some outbreaks indicated rodents as the probable source [19].

Some authors suggested that enhanced transmission of human monkeypox in recent years could be attributed to the cessation of smallpox vaccination campaigns in the 1980s [3,20,21]. It is known that vaccination against smallpox imparted some degree of cross-protection against the other orthopoxviruses, including monkeypox (only for the elderly) [20,21,22].

The emerging changes in the human monkeypox epidemiology need to be considered in order to properly prevent its further spread. In order to do so, it is crucial to increase awareness of healthcare professionals about human monkeypox. This manuscript aims to summarize the epidemiological characteristics of the human monkeypox outbreak in 2022.

## 2. History of Monkeypox

Human monkeypox virus was first identified in 1958 as the causal agent of a pox-like illness during an outbreak among captive cynomolgus macaque monkeys (hence the name monkeypox) in an animal facility at a laboratory in the State Serum Institute in Copenhagen; these monkeys had been imported to Denmark from Singapore for the purpose of research related to the polio-vaccine [23]. Even though later evidence of monkeypox infection was discovered in numerous rodents including rats, mice, and squirrels, the source of the disease remains unknown [24,25]. Hence, the term “monkeypox” may be inappropriate since the exact reservoirs and transmission of the virus are not fully defined [11].

The first human monkeypox case was recognized in Zaire (now the Democratic Republic of the Congo) in 1970, nine months following the country’s smallpox eradication [26]. On 1 September 1970, a 9-month-old child was admitted to hospital with a smallpox-like illness. Specimens from the patient were sent to the WHO Smallpox Reference Centre, Moscow, from which the monkeypox virus was isolated [27]. After recovering, the patient was discharged; however, on October 23 he developed measles and died 6 days later. All other family members had typical smallpox vaccination scars. No further transmission of the infection was evident, either in the village or in the hospital.

Since then, human monkeypox has become endemic in the Democratic Republic of the Congo and has spread to other countries in Africa, mostly in Central and West Africa [28]. The first human monkeypox cases reported outside of Africa occurred in 2003 in the United States of America [29]. In the following years, many cases of human monkeypox were registered [4].

## 3. Epidemiology of Monkeypox

Throughout the past four decades, paradoxically after the global eradication of smallpox in one of the greatest triumphs of preventive medicine, human monkeypox has occurred most often in poor settlements in remote forests in Africa [1,4,30].

### 3.1. Causative Agent 

Monkeypox is a viral disease caused by the monkeypox virus, a member of the Orthopoxvirus genus of the Poxviridae family, Chordopoxvirinae subfamily [31]. In the genus Orthopoxvirus, there are also some other important human and animal viruses including the smallpox virus, vaccinia virus, cowpox virus, etc. In 2001, a genomic comparison of monkeypox and smallpox viruses found 96.3% identity in the central genome region that encodes essential enzymes and structural proteins, while considerable differences were described in the end regions that encode virulence and host-range factors [32,33]. It is known that vaccination against smallpox provides cross-immunity with up to 85% protection against human monkeypox infection [21,34]. Based on genetic, geographic, and phenotypic variations, two distinct genetic clades of the monkeypox virus exist: Clade I, formerly known as the Central African (Congo Basin) clade and Clade II, formerly known as the West African clade; these have epidemiological and clinical differences, with those assigned to the Congo Basin being more virulent and associated with a more severe disease, higher mortality, and more human-to-human transmissability [35,36,37,38,39].

### 3.2. The Course of the Disease

Following infection with the monkeypox virus, an incubation period of approximately 7–12 days and up to 21 days occurs [1,31,40,41]. The smallpox-like prodromal stage lasts 2–4 days and presents with fever, headache, chills, muscle aches, back pain, a general feeling of discomfort, exhaustion and fatigue, and lymphadenopathy. About 1 to 3 days following the onset of fever, a smallpox-like well-circumscribed rash develops, with a rapid typical centrifugal pattern (over the head, body, extremities, including palms and soles, oral mucosa, genitalia). The rash lasts approximately 14–28 days, initially as macules, progressing through papular, vesicular, and pustular phases, and finally crusts. Monkeypox in humans usually occurs as a self-limited disease and typically lasts for 2 to 4 weeks, with complete recovery. Sequelae of monkeypox include respiratory distress, secondary bacterial infections, bronchopneumonia, encephalitis, sepsis, and ocular infections with ensuing loss of vision [42]. Severe illness and worse outcomes occur more commonly among children [43], patients with comorbidity (immune deficiencies including HIV) [44], pregnant women [45], and persons unvaccinated against smallpox (nowadays, those <40–45 years of age could be more susceptible to monkeypox due to the cessation of vaccination against smallpox following the global eradication of the disease) [46]. Infection in pregnancy can result in miscarriage [45]. The case fatality ratio of monkeypox for the West African strain ranged from 0% to 1%, while the Congo Basin strain ranged up to 10% [47].

It is not known how often the asymptomatic infection may occur, but serological surveys gave evidence of asymptomatic human monkeypox infections in persons vaccinated against smallpox and in some unvaccinated persons [48,49,50].

While some authors consider that the first day of the rash marks the beginning of the infectivity period [50], the Centers for Disease Control and Prevention state that sometimes a person may be contagious even in the prodromal period [41]. Patients are most contagious when lesions develop. An infected person is contagious until all scabs have fallen off and new skin covers all the monkeypox spots (lasting for a period of 2 to 4 weeks). Monkeypox virus is highly resistant in dry environments and at high temperatures and persists on infected surfaces for a longer period (about 2 weeks) [41].

### 3.3. Natural Reservoir of Monkeypox Virus

Unlike the variola virus, which infects only humans, the monkeypox virus (as well as other Orthopoxviruses) can infect multiple animal hosts and can spread to humans [24,35,51,52,53,54,55]. Rope squirrels—Funisciuris species, along with giant pouched rats and dormice, were three virus-positive African species associated with the importation of monkeypox into the USA in 2003 [56].

Serological surveys showed that different wild animals can be infected under natural conditions, namely non-human primates (orangutans, chimpanzees, sooty mangabeys, cynomolgus monkeys), rodents (mice, rabbits, squirrels, hamsters, groundhogs), anteaters, prairie dogs, southern opossums, marmosets, and hedgehogs [57,58]. However, although numerous animals were recognized as susceptible to the monkeypox virus, the natural animal reservoir remains unknown [4,37,57,58].

### 3.4. Transmission

The monkeypox virus is transmitted to humans via contact with an infected animal or human, or through contact with contaminated material [1,31,41].

The sporadic occurrence of human monkeypox during 1970–1979 (a total of 54 cases reported) indicated that the monkeypox virus remains in animals and that humans represent only incidental hosts [59,60]. Animal-to-human transmission can occur via broken skin, the respiratory tract by droplets, or mucous membranes [41,60]. A possible risk factor is the consumption of inadequately cooked meat and other animal products originating from infected animals.

Before the 2022 outbreak, animal-to-human transmission through contact with animals was considered the main factor for human monkeypox infections [4,41,61,62,63]. However, in some endemic countries, human monkeypox outbreaks where the index case was apparently infected by an animal were discovered; however, the transmission continued up to a generation [64,65,66].

Human-to-human monkeypox transmission is thought to occur mostly via direct contact with the skin lesions of an infected person (such as infectious rash, sores, scabs) or body fluids, via respiratory droplets (during close and prolonged face-to-face contact), or through contact with recently contaminated objects/fabrics (e.g., bedding, clothing, or towels)/surfaces [1,41]. The direct contact includes intimate contact (sex—oral, anal, and vaginal, or touching the genitals or anus of an infected person), hugs, massage, and kisses, prolonged face-to-face contact, and touching fabrics and objects during sex that a person infected with monkeypox used and which were not disinfected (e.g., bedding, towels, sex toys, and fetish gear).

Transmission through respiratory droplets increases the risk for healthcare professionals, household members, and other close contacts of the monkeypox cases [64,65,66,67,68,69].

Data regarding mother-to-child transmission of monkeypox infection in pregnancy and breastfeeding are limited [41]. Adverse outcomes of mother-to-child transmission of monkeypox virus include neonatal monkeypox infection, miscarriage, stillbirth, and preterm delivery [41,45].

In the 2017 human monkeypox outbreak in Nigeria, a majority of cases were young adult males presenting with genital ulcers, and among them, some cases had concomitant syphilis and HIV infection [70]. However, sexual transmission of human monkeypox is not established, and the role that genital secretions might have in transmission requires further research [41,71,72].

## 4. Epidemiology of Human Monkeypox before the Global Outbreak in 2022

Over the past 50 years, the Democratic Republic of the Congo has been the only country to continuously report cases of human monkeypox, and during the last three decades, the number of reported cases was over 1000 per year [4,73]. In 2020 alone, a total of 6257 suspected human monkeypox cases were reported there [73]. In the first four months of 2022, 1238 new cases were reported in the Democratic Republic of Congo [1]. Most cases of human monkeypox were infected with the Central African clade [4].

Human monkeypox was not reported outside Africa until 2003 when an outbreak occurred in the USA [29,37]. None of the cases in this outbreak (81 identified cases, 40% laboratory confirmed) were attributed to secondary transmission, and there were no deaths. The cases of monkeypox in the US happened after exposure to infected pet prairie dogs. The prairie dogs got the monkeypox virus from infected exotic animals (pouched rats and dormice) imported from Ghana.

Since the 1970s, the rise of human monkeypox has likely occurred due to many factors. These include the surveillance efforts and changes in the environment (deforestation, climate change) and urbanization of areas where the monkeypox virus is maintained in a large number of animal hosts, with an increase in reservoirs or incidental animal hosts. In addition, there is a lack of immunity to the smallpox virus among most people aged 40–45 years or less after the cessation of vaccination against smallpox in the early 1980s. Finally, important factors also include human behavior (such as having contact with live or dead animals/reservoirs, visiting forested or recently deforested areas, hunting, living in the same household with an infected person, sharing with an infected person the same room/bed for sleep, sharing kitchen utensils with an infected person, preparation and consumption of dead bush meat or monkeys), poverty, war, civil conflict and displacements, travel, the exotic pet trade, and health care facilities [14,20,35,67,74,75].

## 5. Epidemiology of Human Monkeypox in the Global Outbreak in 2022

Since early May 2022, for the first time, many outbreaks of human monkeypox have been reported in countries in the European Region, where the disease is not endemic [1,76]. First, between 13 and 16 May 2022, the UK reported six cases of human monkeypox, without any epidemiological links with travel to Africa or imported animals, and with all cases self-identifying as gay, bisexual, or other men who have sex with men [1]. Most confirmed cases of human monkeypox reported a history of travel to countries in Europe and North America. Besides, human monkeypox cases in endemic countries continue to be reported.

**Figure 1:**
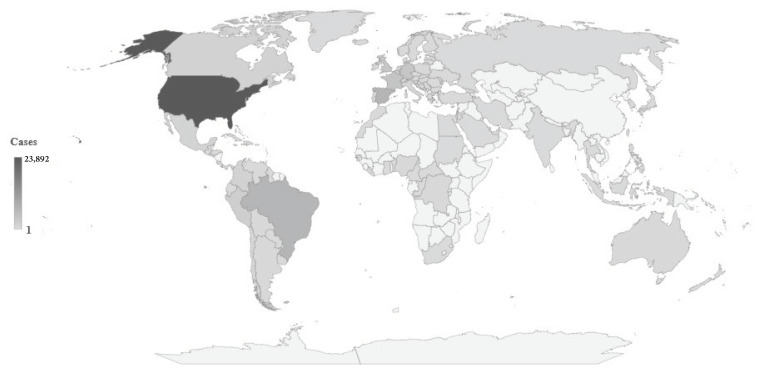
2022 Human monkeypox outbreak global map: Cumulative number of confirmed cases, by countries, data as of 19 September 2022. (Source of data: reference [41]).

Since early May 2022 and as of 19 September, over 62,000 cases of human monkeypox have been reported worldwide, among them almost all in non-endemic countries (Figure 1) [41]. As of 19 September, 44 European countries have reported 24,017 cases, representing 38.5% of all cases reported worldwide in the current outbreak: the largest number was recorded in Spain (6947), followed by France (3898), Germany (3563), and the UK (3552), while one case each was recorded in Turkey and Ukraine. In this outbreak, the largest number of cases (23,892) was reported in the US, representing 38.3% of all reported cases worldwide. Differences in the number of human monkeypox cases by country may be partly explained by differences in population and size of the at-risk populations, socio-economic status, underdiagnosis, and/or underreporting.

For the first time, this outbreak indicated the sustainable chains of person-to-person transmission of human monkeypox that have been reported in Europe [6,76]. In the current outbreak, clinical features that differ from those previously described in available literature were noticed, including the absence of prodrome or very mild prodromal symptoms, a rash that appears before prodrome, a rash that manifests with only a single ulcer or with few lesions, a rash limited only to the anogenital and/or perineal area, and lymphadenopathy with mostly inguinal location [4,8,9]. The severity of the disease is rated as mild to moderate, with approximately 4–10% of cases being hospitalized [1,15,41]. Twenty deaths from monkeypox have been reported in this multi-country outbreak so far, an equal number in Africa and non-endemic countries (mainly due to encephalitis and comorbidities) [41,76]. Although some reports indicated a small number of asymptomatic cases [76], in one cohort study in the UK, known contacts with a person with confirmed infection with monkeypox were registered in only about 25% of cases [77]. Up to now in the ongoing outbreak, there has been no confirmed human-to-animal or animal-to-human transmission. In this outbreak, isolated viruses belong to the West African clade [55,78].

A study conducted at 43 sites in 16 countries reported that 99% of monkeypox cases were men, among whom 98% self-identified as gay or bisexual men, or men who have sex with men [9]. The median age of cases was 38 years, with a range of 18–50 years. Among them, 41% were living with HIV, and in the majority, HIV infection was well controlled. Preexposure prophylaxis was used by 57% of the persons who were HIV negative or did not know their HIV status. In 29% of tested persons, there was a report of concomitant sexually transmitted infections. Although it was not possible to confirm sexual transmission, sexual history was recorded in 95% of cases, with a median number of sex partners in the previous 3 months of 5 (interquartile range 3–15); 20% reported engaging in “chemsex” (sex associated with the use of drugs) in the previous month, and 32% reported attending a sex-on-site event in this month [9].

In the outbreak in Spain [8], 84.1% of cases reported having sex without a condom or having sex with multiple partners within 21 days before the onset of symptoms, 8.1% declared not having sex without a condom, and 7.9% did not answer. Additionally, in the same study, 80.3% of cases were unaware of or reported no contact with a known monkeypox case. Several cases had a history of international travel (including Italy, Portugal, Belgium, Germany, the UK, Peru, etc.) in the month before diagnosis, with no cases reporting travel to Africa. In addition, some cases reported being at a sauna in the city of Madrid or the Gay Pride Maspalomas festival on the Spanish island of Gran Canaria, with numerous private parties also having a significant role (dating through social networks was reported by 56.9% of cases, which led to sexual encounters in private flats, cruising areas, and bars). Although during this outbreak the monkeypox virus was found in samples of seminal fluid of cases and although close physical contact in sexual networks represents an important factor in the ongoing outbreak, more research is necessary to clarify whether human monkeypox can be sexually transmitted via genital fluids [8,9,16,72].

Notably, the lack of vaccination status data was registered in many reports [79]. Among the US monkeypox cases for whom vaccination status was available, 14% reported previously receiving the smallpox vaccine (with 23% receiving one of two doses during the current outbreak, 23% who received pre-exposure prophylaxis at an unknown time before the ongoing outbreak, and 54% who did not give information regarding the time of vaccine administration) [80]. Up to today, 344 cases of monkeypox have been reported among healthcare workers, and among them, some cases of transmission via occupational exposure have been reported in the ongoing outbreak [41]. Globally, persons younger than 40 to 50 years of age are more susceptible to monkeypox due to the cessation of smallpox vaccination campaigns after the eradication of the disease [81]. To prevent monkeypox, two vaccines (JYNNEOS and ACAM2000, both containing a live *Vaccinia virus*) are used: JYNNEOS vaccine is used to protect against both monkeypox and smallpox, while the ACAM2000 vaccine is used to protect against smallpox [81,82]. The immune response after vaccine application is based on the cross-protection between *Vaccinia virus* and *Orthopoxviruses* [31]. In the current outbreak in the USA, persons who have been identified as a close contact of someone with monkeypox, and persons who learn that one of their sex partners in the past 2 weeks has been diagnosed with monkeypox can receive the vaccine [82]. In addition, men who have sex with other men or are a transgender or gender-diverse person who had sex with men in the past 2 weeks could receive the vaccine if they had sex with multiple partners or group sex, or had sex at a commercial sex venue (such as a sex club or bathhouse), or had sex at an event, venue, or in an area where monkeypox transmission is occurring [82]. According to the WHO recommendations, some countries in the European region (the UK and some European Union countries, such as Spain, France, Germany, etc.) are conducting vaccination during the 2022 monkeypox outbreak [81,83].

The WHO assesses the monkeypox risk as moderate globally and in almost all regions, with the exception of the European region and the region of the Americas where the risk is assessed as high [17,81]. The ongoing global outbreak differs from prior outbreaks in a few ways: the unusual magnitude; atypical expansion worldwide; spread in countries where the virus has never appeared; rapid global expansion, predominantly among young men (mostly aged 18 to 44), over 97% of them self-identifying as men who have sex with men involving condomless sex with multiple sexual partners; the role of some superspreading events linked to international gatherings, while asymptomatic infections and lack of or mild symptoms during the prodromal phase of disease make spreading easier; and occurrence of a larger number of secondary cases [1,4,41]. In conclusion, further research is needed to better understand and improve the management of human monkeypox.

## Data Availability

Data is contained within the article.
